# Care for frontline health care workers in times of COVID-19

**DOI:** 10.1590/0037-8682-0358-2020

**Published:** 2020-08-26

**Authors:** Karine Demartini, Vanessa de Mello Konzen, Marcia de Oliveira Siqueira, Gabriela Garcia, Matheus Santos Gomes Jorge, Juliana Secchi Batista, Lia Mara Wibelinger

**Affiliations:** 1 Universidade de Passo Fundo, Programa de Pós-Graduação Stricto Sensu em Envelhecimento Humano, Passo Fundo, RS, Brasil.; 2 Universidade de Passo Fundo, Departamento de Fisioterapia, Passo Fundo, RS, Brasil.

**Keywords:** Coronavirus infections, Occupational health, Pandemics

## Abstract

**INTRODUCTION:**

The spread of the 2019 coronavirus disease (COVID-19) has generated the collapse of health care systems and significant impacts on the health of the workers involved in combatting the disease worldwide.

**METHODS:**

We conducted an integrative literature review focusing on the alternatives implemented to develop care for frontline health care workers in times of COVID-19.

**RESULTS:**

Fifteen articles disclosed the importance of physical and mental care for workers.

**CONCLUSIONS:**

A sensitive view of the health care worker’s care is urgently needed to maintain the quality of health service offered to the population and preserve the health of frontline workers.

Pandemics are outbreaks of infectious diseases that encompass a large geographic area, causing significant demand for medical assistance, supplies rationing, high mortality, and intense overload on health care workers. On March 11, 2020, the World Health Organization updated the status of the outbreak of coronavirus disease (COVID-19), caused by severe acute respiratory syndrome coronavirus 2, as a pandemic. COVID-19 is an infectious disease transmitted through inhalation or contact with infected droplet. Contaminated individuals may develop such symptoms as fever, cough, sore throat, fatigue, and discomfort. The majority of ill patients develop a mild clinical presentation; however, a few cases can progress to a more severe presentation, with the presence of pneumonia or multiple organ failure. Severe cases may lead to death, and the mortality rate is currently between 2% and 3% of infected patients. Given the rapid spread of COVID-19, the following scenario has been a global challenge: health care systems are overloaded in the organizational, clinical, and ethical aspects, with a huge number of exhausted, ill health care workers, resulting in many cases of death^1^ ^-^ ^4^.

In the face of this scenario, a relevant issue emerged: which alternatives have been implemented to develop care for frontline health care workers in times of Covid-19? To find answers, we conducted an integrative literature review using the following electronic databases: Medline/Pubmed, Scielo, Lilacs, and Web of Science. The search terms “coronavirus,” “Covid-19,” “health workers,” “caregivers,” and their synonyms were used. Initially, 57 manuscripts were found. The following inclusion criteria were applied: (1) information regarding protocols, handling, and flows for the protection of health care workers; (2) strategies for health care workers’ care; (3) published until May 2020 in any language. Meanwhile, studies regarding patient handling, therapeutic interventions, past pandemics, or any other health emergencies were excluded. Twenty-one studies were selected for full reading, and 12 articles met the eligibility criteria. Subsequently, we performed a manual search of the selected articles’ reference lists, which identified three more manuscripts. Thus, we collected a final sample of 15 studies.

The selected articles revealed the unique dimension of the pandemic: the COVID-19 scenario is exacerbated when compared with others owing to the current global interconnection of people that accelerated its spread and by the widespread social isolation guidelines that affected almost one-fifth of the world’s population. This amplified scenario generated a collapse in health care systems, leading to drastic decisions aimed to flatten and arrest the disease progression curve, reduce the number of fatal cases, minimize the health system overload, and protect health care workers^2^ ^,^ ^3^ ^,^ ^5^.

With the goal to protect health care workers, alternatives worldwide have recognized the importance of the implementation of guidelines to prepare workplaces to treat COVID-19, by considering it as a new work-related disease. Its inclusion as an occupational disease is due to the easy patient-health care team transmission that can cause a substantial exposure to that biological agent. This risk created intense and rapid changes in the implemented work routine of health care workers, with methodical and strict infection control and consequent increase in interpersonal distance. A reduction in group activities was needed, as well as the inclusion of new procedures. However, such modifications has increased the workload and reduced the break times of workers^2^ ^,^ ^5^ ^-^ ^7^.

The implemented changes in the occupational routines has improved workers’ distress. Nonetheless, workers still needed to make quick and difficult decisions. Therefore, it is fundamental to consider this scenario carefully and allow a division of responsibility such that difficult decisions are made at an institutional level, thereby removing the weight and charge from the frontline workers. To ensure quick and assertive decision making, hospitals managers developed protocols and flows to facilitate the timely actions and decisions of professionals. Moreover, hospitals directors amplified the availability of the recommended individual protection equipment, along with instruction regarding their correct use, to increase the sense of safety and reduce the risk of becoming ill^2^ ^,^ ^3^ ^,^ ^10^ ^,^ ^11^.

In addition, this moment of intense changes is characterized by emotional demands that change in the timeline; data from a multicenter research in China revealed that 73% of frontline health care workers were emotional suffering, 51% reported depression, 45% anxiety, and 36% insomnia. A systematic review confirmed such data, reporting that frontline health care workers present a high prevalence of psychological issues; those who have not presented such problems have a high risk of developing them. Therefore, health authorities need to create protection programs for workers with measures that will assist them into managing the demands they face, strategies for emotional support, and interventions for amplifying their psychological repertory and resilience; health authorities also need to recognize the possible post-traumatic stress disorder in workers involved in the pandemic^3^ ^,^ ^6^ ^,^ ^9^ ^,^ ^12^ ^,^ ^13^.

Added to the global, occupational, and emotional changes are the individual ones, such as fear and uncertainty facing the “new normal” and reduced social and community interaction. Mainly among professionals who are in direct contact with risk group individuals, family support that used to be their backup became a source of distress owing to fear of contamination. These are among the other factors that result in additional overload to health care workers. In the face of the considerable weight on the shoulders of health workers, the big picture needs to be considered in implementing plans and actions for taking care of the fundamental resource that is the human potential^3^ ^,^ ^8^ ^,^ ^9^.

The studies selected suggested that an online mental health service for health care workers could, for instance, identify those at risk of suicide and alert designated volunteers to act according to the situation. This type of service can contribute to the development of emergency actions that could protect the professionals by improving the quality and efficiency of interventions in the assistance provided^14^ ^,^ ^15^.

One of the manuscripts revealed that while seeing to the medical team’s psychological needs, a hospital in Xiangya developed an online intervention plan but this plan failed in every measure taken. This hospital also designed other attempts to reduce group stress, among them resting accommodation and temporary family isolation, in which family members could share in the worker’s routine, thereby minimizing the latter’s concern; specific training; and recreational activities and personal support provided by psychologists. These latter actions were successful and could be reproduced^12^.

Feelings such as fear, uncertainty, and stigmatization are common in any biological disaster and may act as barriers to the health care team’s interventions. Maintaining the worker’s mental health is essential to controlling infectious diseases, although the best approach to this situation is unclear. The development and implementation of evaluations, support, treatment, and mental health services are shown to be crucial points and vital to the health care response to the COVID-19 outbreak. If these psychological issues are not solved in an efficient manner, they may not only lead to an immunity decline and increase the chances of infection but also have an adverse impact on the quality and safety of the health assistance system^4^ ^,^ ^12^.

A review study with meta-analysis suggested that the redistribution of staff to care for patients positive for COVID-19 should be voluntary where possible. The authors identified that the risk factors for psychological distress include the following: being younger, inexperienced, parents to dependent children and quarantined, having an infected family member, lacking practical support, and stigma. Strategies that may be easily employed to minimize the psychological distress of health professionals are as follows: clear communication, access to adequate individual protection, sufficient rest, and practical and psychological support; such strategies are associated with mortality reduction^13^.

In view of the above and the current global health scenario, the alternatives already implemented reinforce the need for managers to adaptable when implementing strategies of care and for the physical and psychological protection of frontline health care workers against the pandemic. 
